# Corpora amylacea negatively correlate with hippocampal tau pathology in Alzheimer’s disease

**DOI:** 10.3389/fnins.2024.1286924

**Published:** 2024-02-29

**Authors:** Julian D. Dallmeier, Ryan Gober, Regina T. Vontell, Ayled Barreda, Daniel A. Dorfsman, David A. Davis, Xiaoyan Sun, Daniel Brzostowicki, Illiana Bennett, Susanna P. Garamszegi, Connor M. Wander, Todd Cohen, William K. Scott

**Affiliations:** ^1^Brain Endowment Bank, Department of Neurology, University of Miami Miller School of Medicine, Miami, FL, United States; ^2^Department of Neurology, Evelyn F. McKnight Brain Institute, University of Miami Miller School of Medicine, Miami, FL, United States; ^3^John P. Hussman Institute for Human Genomics, University of Miami Miller School of Medicine, Miami, FL, United States; ^4^Department of Neurology, University of North Carolina at Chapel Hill, Chapel Hill, NC, United States

**Keywords:** corpora amylacea, Alzheimer’s disease, neuropathology, neurofibrillary tangles, *APOE*, brain, wasteosomes

## Abstract

**Introduction:**

Severity and distribution of aggregated tau and neurofibrillary tangles (NFT) are strongly correlated with the clinical presentation of Alzheimer’s disease (AD). Clearance of aggregated tau could decrease the rate of NFT formation and delay AD onset. Recent studies implicate corpora amylacea (CA) as a regulator of onset or accumulation of tau pathology. Normally, CA clear brain waste products by amassing cellular debris, which are then extruded into the cerebrospinal fluid to be phagocytosed. The proper functioning of CA may slow progression of AD-associated NFT pathology, and this relationship may be influenced by amount and distribution of phospho-tau (pTau) produced, age, sex, and genetic risk.

**Objective:**

The goal of this study was to determine if CA size and number are associated with hippocampal location and local pTau severity while accounting for variations in age, sex, and genetic risk.

**Methods:**

Postmortem brain hippocampal tissue sections from 40 AD and 38 unaffected donors were immunohistochemically stained with AT8 (pTau) and counter stained with periodic acid Schiff (PAS). Stained sections of the CA1 and CA3 regions of the hippocampus were analyzed. The percent area occupied (%AO) of CA, pTau, and NFT was calculated. Pairwise comparisons and regression modeling were used to analyze the influence of age, pTau %AO, and genetic risk on %AO by CA in each region, separately in donors with AD and unaffected donors.

**Results:**

CA %AO was significantly higher in the CA3 region compared to CA1 in both groups. A significant negative correlation of CA %AO with both pTau %AO and neurofibrillary tangle %AO in the CA3 region of AD brain donors was found. Regression analysis in the CA3 region revealed a significant negative association between CA with both pTau and age.

**Conclusion:**

We found an increase of CA in the CA3 region, compared to CA1 region, in AD and unaffected donors. This may suggest that the CA3 region is a hub for waste removal. Additionally, the negative correlation between %AO by CA and NFT in the CA3 region of the hippocampus in donors with AD suggests CA could play a role in AD pathologic progression by influencing tau clearance.

## 1 Introduction

Alzheimer’s disease (AD) is the most prevalent cause of dementia in the developed world and while the principal pathology behind the disease is well characterized, the molecular mechanisms leading to the progression of the pathology are not well understood. Insight on the mechanisms of pathologic progression may promote the development of effective treatments for AD. AD is neuropathologically classified as a protein aggregation disorder characterized by the presence of amyloid beta (Aβ) plaques and tau-containing neurofibrillary tangles (NFT). These protein aggregates build up over time and correlate with progressive neurodegeneration (Braak and Braak, 1995; Montine et al., 2012). The severity and distribution of aggregated tau and neurofibrillary tangles (NFT) are strongly correlated with the clinical presentation of Alzheimer’s disease (AD) (Bierer et al., 1995). Unregulated protein aggregation initiates a cascade of detrimental effects on the nervous system, including inflammation and neuronal loss (Gómez-Isla et al., 1997; Akiyama et al., 2000). Given that micropathological protein aggregation is the primary driver in progressive neurodegeneration in AD, understanding the brain’s innate protein clearance mechanisms and how their dysfunction might influence such aggregation becomes essential to fully understanding disease pathogenesis. Dysfunctional protein clearance may lead to faster pathological progression due to unchecked protein aggregation, leading to worse cognitive outcomes.

Recent research on protein aggregate clearance has identified potential mechanisms of resistance (Iliff et al., 2012). The glymphatic system has been shown to flush out extracellular amyloid beta deposits (Iliff et al., 2012), but little is known about the removal of pTau. Efficient removal of pTau may be critical to buffering the formation of NFTs. NFTs are formed by aggregations of hyperphosphorylated tau, which can be visualized with an AT8 antibody. Understanding the brain’s innate mechanisms for pTau removal is essential to understanding the pTau life cycle and identifying potential therapeutic targets.

An alternative to the clearance of tau through the glymphatic system is corpora amylacea (CA)-mediated waste clearance, a mechanism present in the central nervous system and other tissues. CA, also known as wasteosomes (Riba et al., 2022a,b, 2023), have recently been linked to the glymphatic system (Riba et al., 2022b). CA are granular bodies ranging from 10 to 50 μm that accumulate waste products throughout the brain (Cavanagh, 1999; Navarro et al., 2018; Riba et al., 2019). CA are thought to be generated mainly by astrocytes in response to cellular stress, but the exact mechanism of generation is unknown (Cavanagh, 1999). CA are comprised of polysaccharides, mainly glycogen and proteins (Riba et al., 2021). CA are unspecific in their cargo sequestration, and studies have found a range of contents, including proteins, organelles, macromolecules, viruses, and other cellular waste products (Augé et al., 2018). CA have been associated with aging and neurodegeneration (Pisa et al., 2016, 2018), but the main cause and effect remain unknown (Rohn, 2015). CA were first noted by J. E Purkinje in 1837 (Catola and Achúcarro, 1906), but the first known paper was published by Virchow (1854) and Eastman (1897). This was followed by a lull in CA research until a seminal study by Riba et al. (2019) demonstrated that CA amass waste products and are released into the CSF where they are expelled to the cervical lymph nodes (Riba et al., 2019). A complete history of CA has been detailed by Riba et al. (2021). Subsequent studies investigated the contents of CA to determine if CA play a role in AD pathology. A study by Wander et al. (2020) examined the contents of CA and found that some stain positive for the tau-1 antibody, which stains hypo-phosphorylated tau at the tau-1 epitope, thus identifying tau that is hypo-phosphorylated at S202 and T205. These tau-1 positive CA were colocalized to reactive glia. A follow-up study by Riba et al. stained for tau-5, which stains total tau irrespective of phosphorylation, and found tau-5 positive CA in AD patients but not in unaffected controls (Riba et al., 2023). Since tau-5 is not specific to pTau, it is not clear whether CA contain hyperphosphorylated tau, whether they only uptake hypo-phosphorylated tau, or if they have tau dephosphorylation capabilities. Nonetheless, after much debate about the contents within CA, it is now clear that tau is a *bona fide* component of these structures that is potentially targeted for CA-mediated clearance.

Subsequently, we described a bimodal distribution pattern of CA across semi-quantitative NFT severity levels (following Braak AD staging) in the dentate gyrus of post-mortem brain tissue samples (Braak and Braak, 1995; Wander et al., 2022). We found increased CA in the dentate gyrus with rising Braak NFT stages I – III, with a opposite trend across stages IV – VI, with CA levels gradually declining as Braak stage progressed (Wander et al., 2022). This result suggests a quadratic relationship between CA and Braak NFT severity, with the highest levels seen at intermediate NFT severity (stage III) (Wander et al., 2022). In the present study, our goals were to determine whether CA populations are consistent across other hippocampal subregions implicated in early and late AD (CA1 and CA3). We sought to determine if the amount of CA was associated with the amount of pTau and NFT in donors with AD, while controlling for genetic risk of AD, age, and sex. Our use of quantitative methods to evaluate AD-associated neuropathologic change improves our understanding of CA’s role in mediating AD pathology. Further understanding the association of CA and AD pathology across different hippocampal regions will provide a more detailed view of CA as a potential waste clearance pathway in the onset and progression of AD.

## 2 Methods

### 2.1 Postmortem human tissue

**Figure 1:**
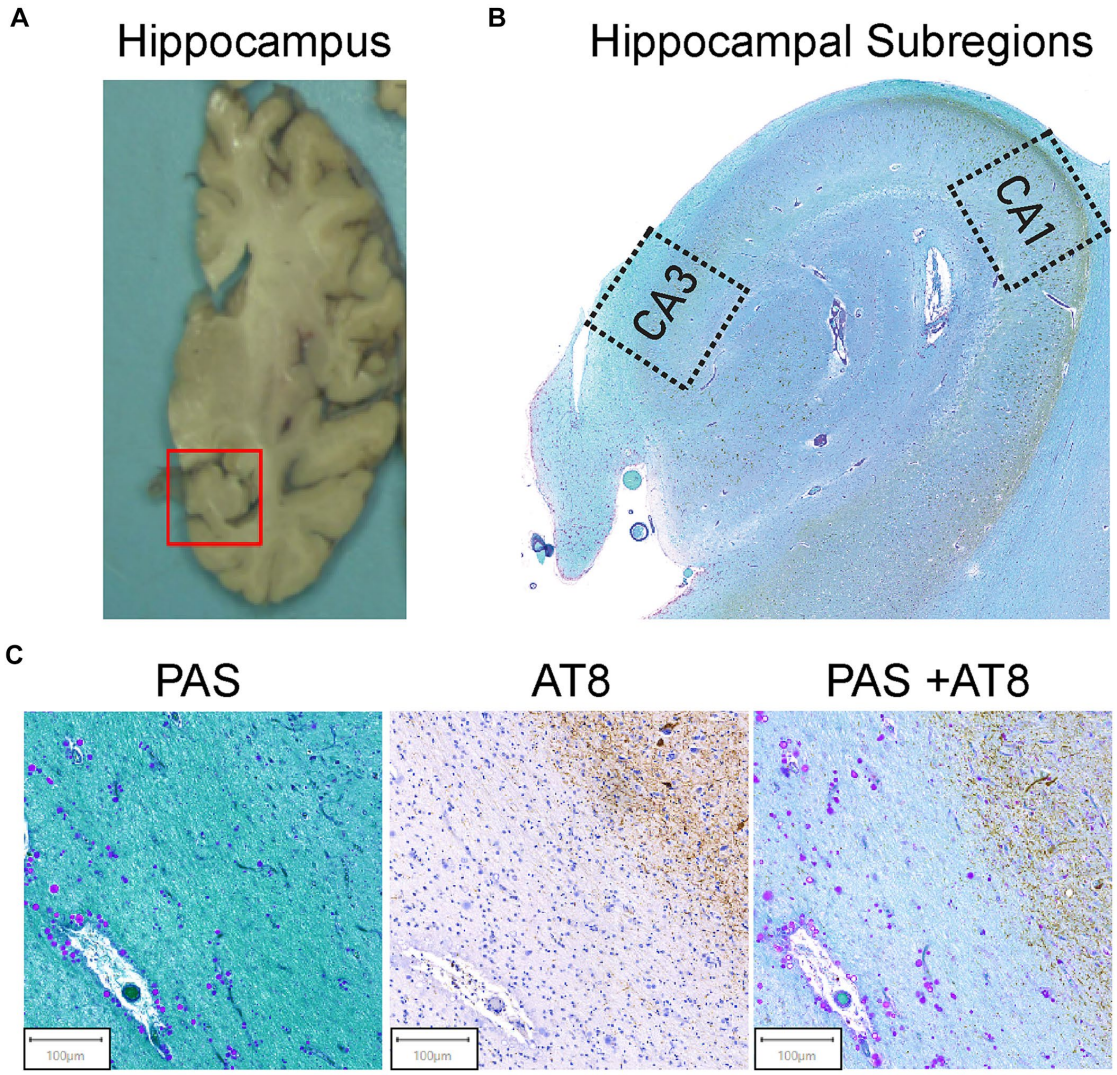
Hippocampal region selection and representative images of tissue staining. **(A)** Coronal view of the brain’s right hemisphere. The red box identifies the hippocampal region. The hippocampus was cut from the brain and embedded in paraffin. **(B)** The Cornu Ammonis 1 (CA1) and Cornu Ammonis 3 (CA3) regions were isolated from the hippocampus as outlined. Average ROI size ranged from 4 to 6 mm^2^. **(C)** AT8 and PAS Tissue Staining. Representative images from the tissue staining protocol for all samples. Periodic acid Schiff (PAS) for corpora amylacea (left). CA can be identified by their spherical morphology and the purple stain from the PAS. AT8 was used for pTau (middle). AT8 stains pTau dark brown, and brown teardrop shaped structures are pTau aggregates in the form of NFTs. Double staining of PAS + AT8 was used for pTau and CA in the same field of view (right). CA and pTau aggregates can be visualized by purple circles and brown punctate and teardrop formations, respectively.

Pre-mortem informed consent for research and post-mortem authorization for retention of brain tissue for research were obtained in accordance with the University of Miami Institutional Review Board (IRB) protocol 19,920,348. The left hemisphere was frozen, and the right hemisphere was fixed in 10% formalin, sliced, and then sampled in paraffin embedded blocks (Leica Biosystems). Clinical characterization of brain donors consisted of a combination of periodic pre-mortem cognitive screening tests (TICS-m on a subset of donors; Brandt et al., 1988) and a review of medical records from primary care physicians or neurology or psychiatry specialists on all donors (XS, AB). Based on all available clinical information and a detailed neuropathological examination of standard brain regions, a consensus diagnosis was determined for each donor. Neuropathological evaluations were carried out by neuropathologists who provided detailed neuropathologic reports, including Braak NFT stage and B score (Montine et al., 2012). Paraffin-embedded tissue sections were taken from the mid hippocampal region, including the Cornu Ammonis 1 (CA1) and 3 (CA3) regions (Figure 1). Serial sections of the paraffin-embedded tissue blocks were sliced at 8 μM thickness using a Leica RM2245 microtome (Leica Microsystems Ltd., San Diego, *CA.*) and fixed to glass slides.

### 2.2 Immunohistochemistry

Phospho-tau was identified using the AT8 mouse monoclonal antibody staining procedure. Slides were placed in an oven set to 60^o^ C for 1 h to allow the paraffin to melt. They were then deparaffinized in xylene, rinsed in ethanol (100, 95, 70%) and rehydrated in water. The endogenous peroxidase activity was quenched by placing slides in 3% hydrogen peroxide (H_2_O_2_) for 10 min, after which they were rinsed with 1X PBS. The sections were immersed for 5 min in 98% formic acid, washed in cold water, and finally rinsed in 1X PBS. The segments were blocked for 20 min in 5% goat serum (Vector Laboratories) and then overnight incubated at 4°C in PBS in a solution of a mouse anti-phospho-Tau antibody (Ser202, Thr205; 0.4 μg/ml; Thermo Fisher Chemicals; Waltham, MA). The next day, sections were exposed to secondary antibodies of biotinylated horse anti-mouse IgG (15 μg/ml; Vector Laboratories; Newark, CA) to PBS for 1 h, followed by a complex of amino acids (1:200, ABC; Vector Laboratories) for 1 h. The reactions were visualized for 10 min with 3,3′-diaminobenzidine (MilliporeSigma; Burlington, MA).

### 2.3 Histochemistry

After IHC was completed, the PAS stain was performed to evaluate and quantify *CA.* Slides were oxidized in a 0.5% periodic acid solution for 5 min. Slides were then washed with distilled water and placed into Schiff reagent (MillporeSigma) for 15 min at room temperature. To develop the PAS reaction, slides were then washed in running tap water for 10 min. A 16% working light green solution was used to counterstain the slides. Slides were then dehydrated with 95 and 100% alcohol, cleared with xylene, mounted with resin and cover slipped.

### 2.4 Feature quantification

Stained slides from 38 unaffected controls and 40 donors with confirmed AD were scanned and digitized using a Motic Easy Scanner with a zoom of 40x. A total of three slides were analyzed and averaged for each brain tissue sample (120 AD slides and 114 unaffected control slides). Scans were initially analyzed by a single experimenter who was blinded to the affection status of the donors. Prior to analysis, donor IDs were assigned a random numerical code to ensure blinding. Additionally, a second reader performed quantifications on a subset of 10% of the scans to determine inter-experimental variability, which resulted in similar quantifications. Both experimenters adhered to a standardized protocol using algorithmic feature quantification to ensure consistency, and quantifications were correlated to determine consistency (*r* = 0.98). However, it is important to note that experimenters were aware of the type of stain used and the region of interest, which were identifiable from the images. Digitized scans were visually inspected with QuPath (Bankhead et al., 2017) for tissue tearing, immunoreactivity, and proper stain quality. AT8, PAS, and AT8/PAS double staining can be visualized in Figure 1C. The AT8 reaction, which stains phospho-tau, resulted in dark brown staining of pTau-positive cells (Figure 1C). The PAS stain resulted in a pink/purple color for polysaccharides, the major component of CA (Figure 1C). QuPath was used for ROI (4-6 mm^2^) extraction (CA1 and CA3 hippocampal regions) and for .tiff filetype conversion. ROI files were imported into ImageJ (Schneider et al., 2012) for feature extraction and quantification. The CA1 and CA3 regions were isolated from each slide as seen in Figure 1B. ROIs were extracted as described previously (Vontell et al., 2022). In the CA1 and CA3 regions, CA were annotated and quantified using a color thresholding algorithm in ImageJ. This technique isolates features of interest by thresholding specific hue-saturation-brightness (HSB) values. Using color thresholding, the HSB values for positively stained CA (H: 175-240, S: 115-255, B: 215-255) were isolated. Once isolated, a binary mask was applied, excluding all other HSB values. Once CA were marked and isolated by color thresholding, CA were visually reviewed for confirmation. Using the particle analyzer feature of ImageJ, the number and area of all CA were measured. HSB filtering was also used to quantify pTau and NFTs (H: 60-215, S: 40-185, B: 0-105). For the total pTau measure, all positively stained tau was included, and the number and area of stained features were calculated. For NFT pathology, smaller aggregations of pTau were filtered out, larger teardrop NFTs were visually confirmed, and the number and area were extracted. An average of 83 CA, 38 NFTs, and 3,162 pTau events were counted. Then, the number and area of CA, pTau, NFTs, and total ROI area were imported into R version 4.1.3 (R Core Team, 2021) to calculate the area occupied (%AO). The stained features were quantified using a percentage of area occupied formula generated in R. The %AO formula takes the sum of the areas of all positively stained features in a region and normalizes by area of the ROI. The formula is as follows:


$$\%AO=\left(\frac{\mathrm{sum}\left(\mathrm{area}\;\mathrm{of}\;\mathrm{stained}\;\mathrm{features}\right)}{ROI\;\mathrm{area}}\right)*100$$

The %AO metric captures both size and number of CA and normalizes based on the ROI.

### 2.5 Genetic risk assessment

The risk of AD potentially attributable to genetic variation was assessed by calculating a weighted genetic risk score (GRS) from genome-wide genotyping data. Genotypes were generated by the Alzheimer’s Disease Sequencing Project (ADSP) Follow-Up Study at the UM Hussman Institute for Human Genomics. DNA extracted from brain tissue was genotyped using the Illumina Genomic Screening Array (GSA). Genetic data were quality controlled (Anderson et al., 2010) and imputed using the TOPMed Imputation server (Fuchsberger et al., 2015; Das et al., 2016; Taliun et al., 2019). Prior to imputation, SNPs that contained excess missingness above 5% were removed. SNPs with significant evidence of deviation from Hardy–Weinberg equilibrium were also excluded (*p* < 0.0001), leaving 298,509 SNPs for imputation. Monomorphic sites (8,412) and SNPs with low call rates (703) were excluded during imputation. A total of 9,185 sites were excluded after imputation and QC. SNPs that were imputed with a minor allele frequency (MAF) less than 1% were retained if they had an imputation quality score (*R*^2^) greater than 0.8. For SNPs with a MAF of 1% or greater, a *R*^2^ threshold greater than 0.3 was applied for inclusion. After imputation and quality score filtering, a total of 1,422,149 SNPs were used for analysis. To calculate genetic risk score (GRS), we employed PRSice-2.3.5 (Choi and O’Reilly, 2019). The GRS score consisted of 76 out of the 83 variants from Bellenguez et al. (2022) with an imputation quality score (R-squared) higher than 0.8. The Bellenguez GRS did not contain *APOE*. *APOE* was assessed as an independent variable consisting of *APOE* e4 allele counts. Using PRSice2, the GRS was calculated using an additive model from the risk allele imputed dosage weighted by the log odds ratio for each SNP where S = effect size (log odds ratio) of the ith allele and G = effect allele dosage i (between 0-2). The model can be seen in the following.


$${GRS}_{j}=\sum S_{i}\;x\;G_{ij}$$

Since *APOE* is by far the strongest genetic risk factor for the development of AD and is not contained in the Bellinguez GRS, genetic risk was divided into two variables: (1) *APOE* (coded by counting the number of *APOE* e4 alleles present, not weighted by effect size); and (2). GRS for the remaining SNPs.

### 2.6 Data analysis

Intrasubject variability was accounted for by assessing correlations between replicate slides along the rostro-caudal axis. The %AO showed a strong correlation (*r* > =0.98) among the measures of CA, pTau, and NFT within each brain, therefore an average %AO across all slides was used in analysis. CA associations were performed in both AD and unaffected brain donors. We used a Wilcoxon matched pairs signed rank test for pairwise comparisons of variables within each group (AD and unaffected control). To determine if CA %AO was associated with age in donors with and without AD, we employed a Spearman nonparametric correlation between CA %AO and Age in both AD and unaffected brain donors. Additionally, we used a Spearman nonparametric correlation to determine the relationship between pTau and NFT %AO with CA %AO in the CA1 and CA3 hippocampal regions. A stepwise regression model was employed to assess CA association with diagnosis while adjusting for genetic risk of AD, age, and sex. Next, the association between CA and pTau was assessed in AD brain donors. A stepwise generalized linear regression model was fit to assess the association between CA %AO and pTau %AO while adjusting for age (in years), sex, *APOE* e4 allele count, and GRS as covariates. Predictive performance was assessed by Total Sum of Squares (TSS) and Akaike Information Criterion (AIC), and variables were excluded based on significant changes of the model fit as indicated by change in AIC. The model used pTau to represent pathogenic tau due to the high correlation with NFT %AO. *p* < 0.05 was used to determine statistical significance.


$$CA\%AO=\alpha +\beta_{1}\;\mathrm{pTau}\%AO+\beta_{2}\;\mathrm{APOE}+\beta_{3}\;GRS+\beta_{4}\;\mathrm{Age}+\beta_{5}\;\mathrm{Sex}$$

All group comparison results are discussed as mean and standard error of the mean (SEM). All plots and statistical analyses were done in R version 4.1.3 (R Core Team, 2021) and GraphPad Prism 9.0 (GraphPad Software, San Diego, CA). Stepwise regression modeling was performed in R.

## 3 Results

### 3.1 Cases with AD pathology had lower age at death, higher genetic risk, and reduced brain weight

Complete demographics and neuropathologic staging on the unaffected control and AD cohorts is provided in Supplementary Tables S2, S3. Human brain tissue samples from 38 donors (Braak 0-III, age 86 ± 11) with neuropathologic age-related changes (unaffected controls) and 40 donors with confirmed AD neuropathological changes (Braak IV-VI, age 79 ± 12) were selected from the Brain Endowment Bank at the University of Miami Miller School of Medicine (Supplementary Tables S2, S3). Complete demographic information for the unaffected controls and AD brain donors is provided in Supplementary Tables S2, S3. All AD donors had Braak NFT stages of IV-VI. The cohort consisted of non-Hispanic white individuals. In the unaffected controls, 33 donors had no tau pathology (Braak 0), four had low levels of tau pathology (Braak I-II), and one donor had moderate tau pathology (Braak III). In the AD group, there were 14 males and 26 females; this distribution reflects the higher incidence of AD in females. In the unaffected control group, there were 19 males and 19 females. Braak NFT stage was limited to 0-II in the unaffected control group. On average, AD brain donors exhibited increased frequencies of *APOE* e4 carriers: 15% possessed two *APOE* e4 alleles, 40% had one, and 45% had none. In contrast, the unaffected controls showed 3, 31, and 66%, respectively, for the same allele distributions. Additionally, brain weight in the AD group (1,072 ± 22 g) was significantly lower compared to the unaffected controls (1,241 ± 27 g) with a difference of 176 grams (*p* < 0.0001).

### 3.2 Amount of corpora amylacea varies by hippocampal subregion in individuals with and without AD

Since each hippocampal subregion is diverse in AD-related protein deposition patterns, we sought to determine if the total amount of CA differs in the CA1 and CA3 hippocampal subregions and whether this variation differs by disease status. In individuals with AD, pTau deposits generally occur in the CA1 region early in the disease process and eventually spread to the CA2 and CA3 regions later in the disease (Furcila et al., 2018). However, there does appear to be variability in tau deposition patterns (Vogel et al., 2021). The CA1 and CA3 regions were chosen since CA1 is impacted earlier in the disease and CA3 is impacted later, providing information on CA variation throughout disease progression. To determine if there were changes in the total area occupied by CA in different hippocampal regions, the CA1 and CA3 regions from digitized immunohistochemical slides from AD and unaffected control brain donors were isolated. CA quantifications were performed using % area occupied (%AO) for each region.

**Figure 2:**
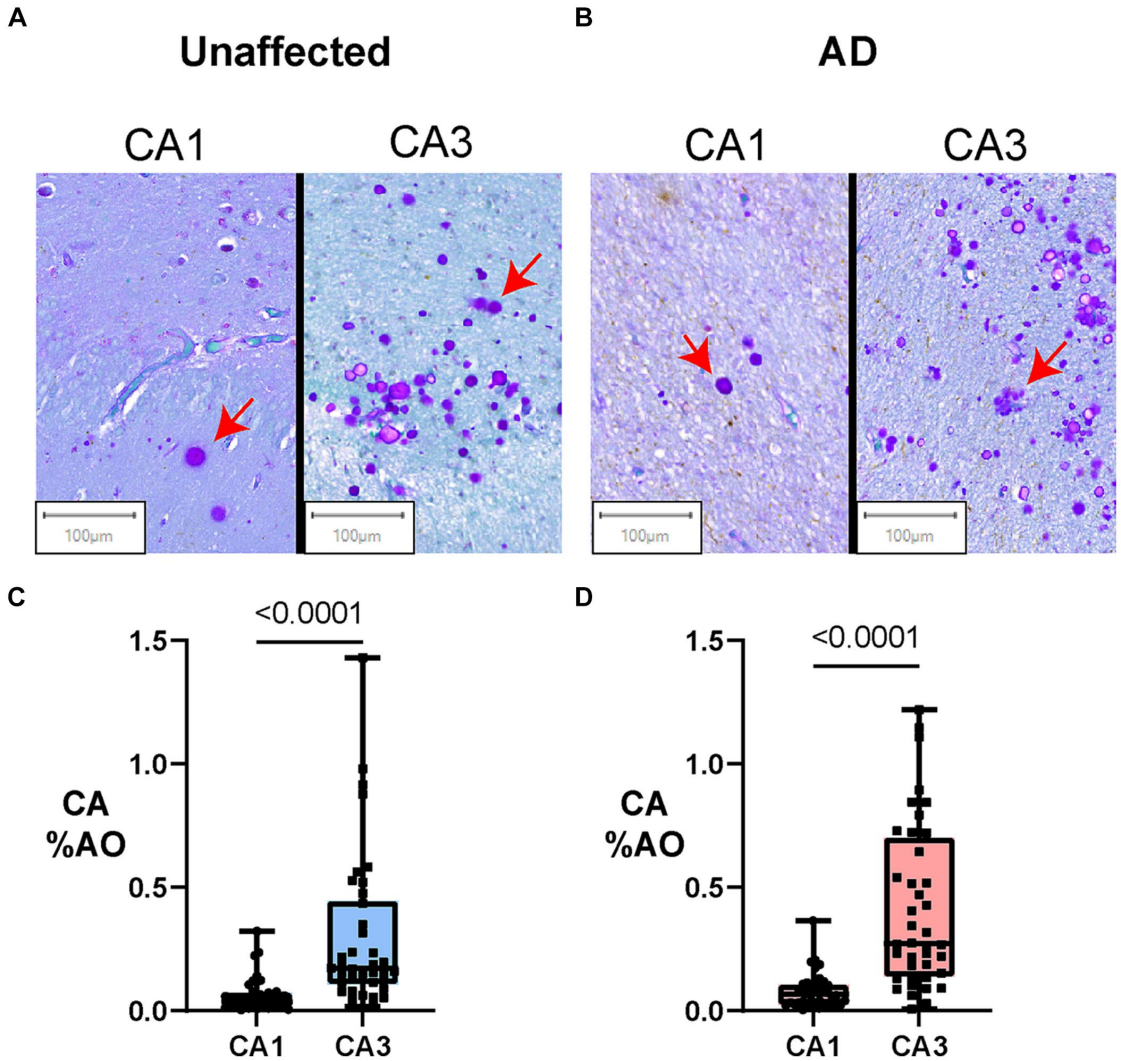
Amount of Corpora Amylacea varies by hippocampal subregion in individuals with and without AD. Box plots represent the distribution of CA levels in the CA3 and CA1 regions of the hippocampus in AD and unaffected controls. The central line indicates the median, with the borders of the box depicting Q1 and Q3 quartiles. Whiskers extend to the lowest and highest values within a 1.5 x interquartile range. **(A)** Digital slide scans of PAS+ CA in the CA1 and CA3 regions of unaffected brain donors. CA are spherical, purple-stained structures that often aggregate in clusters. More numerous CA can be found in clusters in the CA3 region in both AD and unaffected controls. **(B)** Digital slide scans of PAS+ CA in the CA1 and CA3 regions of AD brain donors. More CA can be seen in the CA3 region when compared to the CA1. **(C)** Hippocampal region comparisons in unaffected brain donors (Paired Two-tailed Wilcoxon matched-pairs signed rank test). CA %AO was significantly higher in the CA3 region compared to CA1 in unaffected brain donors (0.29, *p* = <0.0001). **(D)** Hippocampal region comparisons in AD brain donors (Paired Two-tailed Wilcoxon matched-pairs signed rank test). CA %AO was also significantly higher in the CA3 region compared to CA1 in AD donors (0.33, *p* = 0.0001).

Figure 2 presents regional differences in CA in unaffected donors and people with AD. Representative images of CA in the CA1 and CA3 regions can be seen in unaffected (Figure 2A) and AD (Figure 2B) brain donors. As demonstrated in Figure 2, we found that in AD donors, CA %AO was significantly higher in the CA3 region (0.41 ± 0.05) compared to CA1 (0.08 ± 0.01) region, resulting in a significant mean difference of 0.33 (*p* < 0.0001). A similar pattern was seen in the unaffected donor group where the CA3 region (0.30 ± 0.05) contained a higher CA %AO than the CA1 region (0.07 ± 0.01), with a significant mean difference of 0.29 (*p* ≤ 0.0001). CA aggregated most in the CA3 region in clusters close to the ventricular lining. These data demonstrate that CA occupy a larger area of the CA3 region (compared to CA1) in the hippocampus of individuals with and without AD.

### 3.3 The relationship between CA and age in unaffected donors vs. donors with Alzheimer’s disease

**Figure 3:**
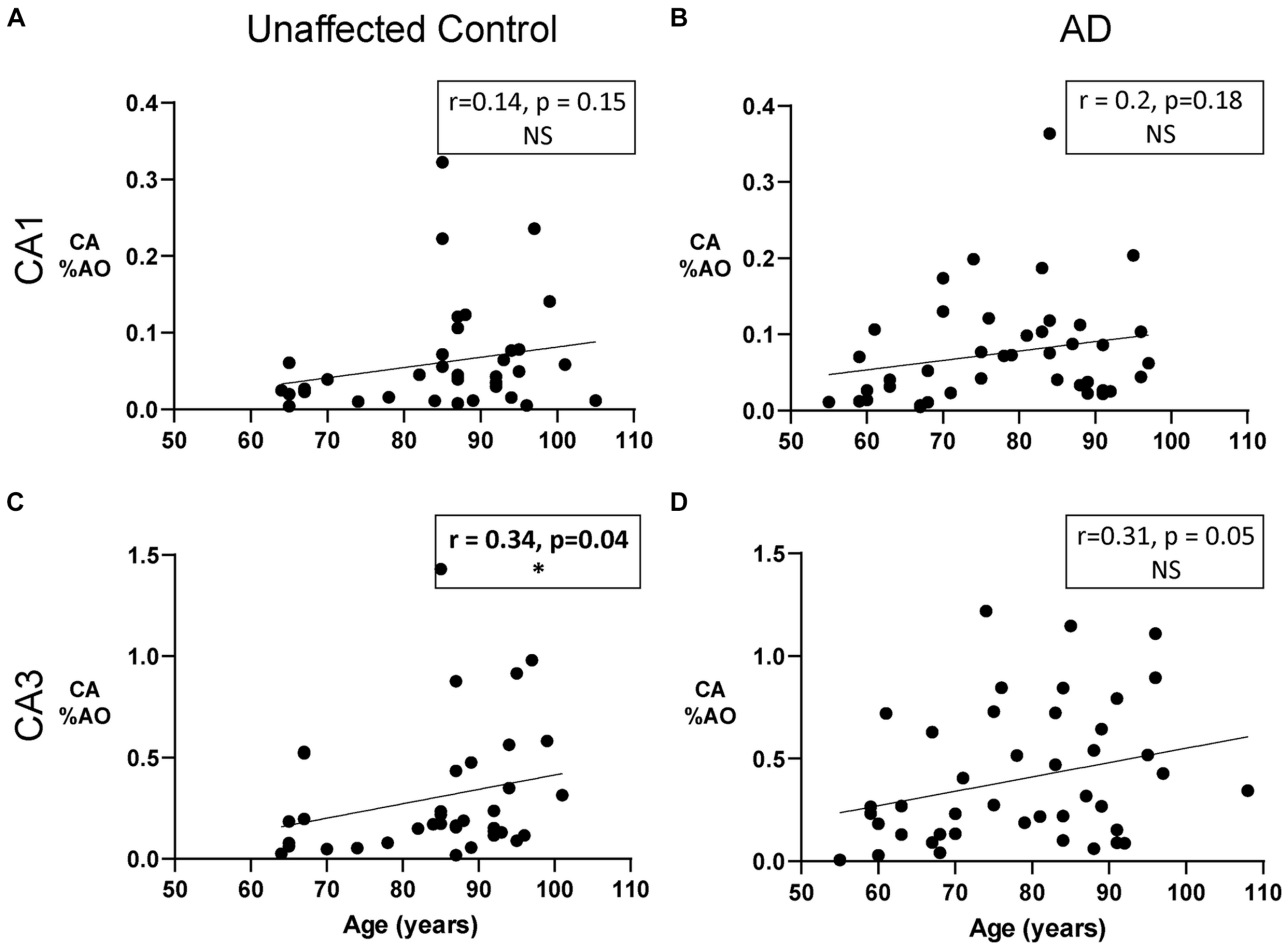
The Relationship between CA and age in unaffected donors vs. donors with Alzheimer’s disease. **(A)** Correlation of CA %AO with age in the CA1 region of unaffected donors. No relationship was found. **(B)** Correlation of CA %AO with age in the CA1 region of AD patients. No relationship was found. **(C)** CA %AO was significantly correlated with age in the CA3 region of unaffected donors (*r* = 0.34, *p* = 0.04). **(D)** Correlation of CA %AO with age in the CA3 region of AD patients. No relationship was found. *R* values represent the correlation coefficient and statistical significance is indicated in bold with a * (0.05 level). Axis ranges varied by region and disease status to visualize all data points.

We next sought to determine whether the area occupied by CA in the CA1 and CA3 regions is associated with age in donors with and without AD. Previous research suggests a linear increase in CA number with age, with their first appearance around age 30–50 followed by a steady increase in size and number (Cavanagh, 1999). We found a significant positive correlation between age and CA %AO in the CA3 region of the hippocampus in unaffected donors (Figure 3C; *r* = 0.34, *p* = 0.04) but not in donors with AD (Figure 3D; *r* = 0.31, *p* = 0.05). Thus, donors with AD showed similar, but not statistically significant, correlations. No significant correlations were found in the CA1 region of either group (Figures 3A,B).

### 3.4 Amount of corpora amylacea is negatively associated with area occupied by NFTs in the CA3 region of donors with AD

**Figure 4:**
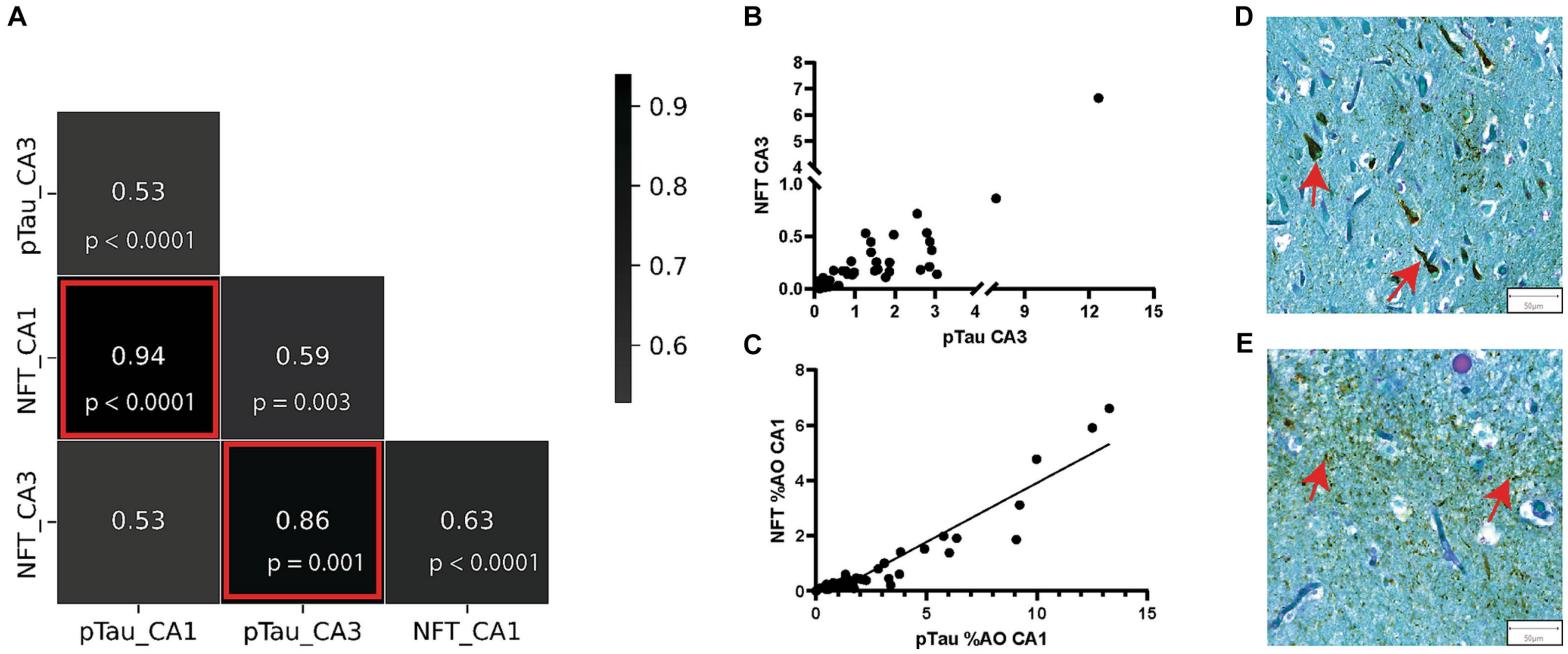
pTau and NFT %AO Are Positively Correlated in AD Brain Donors. **(A)** Correlation matrix consisting of %AO measures of pTau and NFT in the CA1 and CA3 regions. The strongest correlations were seen between the NFT and pTau measures in both CA1 and CA3. These correlations are highlighted by red boxes and are visualized in scatter plots in **(B,C)**. **(B)** Scatter plot of NFT %AO vs. pTau %AO in the CA3 hippocampal subregion. There is a positive correlation (*r* = 0.82, *p* < 0.0001). Note a discontinuous axis was used to visualize the distribution. **(C)** Scatter plot of NFT %AO vs. pTau %AO in the CA1 hippocampal subregion. Black line denotes line of best fit for the positive correlation (*r* = 0.86, *p* < 0.0001). **(D)** Representative image of NFT formation in the CA3 region of the hippocampus in an AD brain donor. Red arrows identify NFTs (50 μm). **(E)** Representative image of pTau deposition in the CA3 region of the hippocampus in an AD brain donor. pTau stains brown and can be seen throughout the image. Red arrows identify pTau clusters (50 μm).

We previously reported that increasing numbers of CA are associated with intermediate stages of NFT pathology in the dentate gyrus in AD (Wander et al., 2022). We found a quadratic distribution pattern of CA area occupied across Braak stages, with a peak of CA at Braak III (Wander et al., 2022). We next aimed to further explore the relationship between area occupied by CA and area occupied by pTau and NFT in the CA1 and CA3 regions of the hippocampus. The pTau metric measured total pTau (Figure 4E), regardless of shape and size whereas the NFT metric consisted of only teardrop shaped NFT pathology (Figure 4D). First, to confirm the expected relationship between pTau and NFT, we constructed a correlation matrix consisting of pTau and NFT measures in the CA1 and CA3 regions. As expected, all variable combinations were significantly correlated (Figure 4A), with pTau and NFT correlations within each region being the strongest. NFT and pTau were strongly correlated in the CA3 (*r* = 0.86, *p* < 0.0001, Figure 4B) and CA1 (*r* = 0.94, *p* < 0.0001, Figure 4C) region of the hippocampus. Additionally, the CA1 region contained a higher amount of pTau (*p* = 0.008) and NFTs (*p* = 0.0001) than the CA3 region of donors with AD (Supplementary Figure S1). Since pTau is a more inclusive measure of hyperphosphorylated tau aggregation, it was used for regression model building.

**Figure 5:**
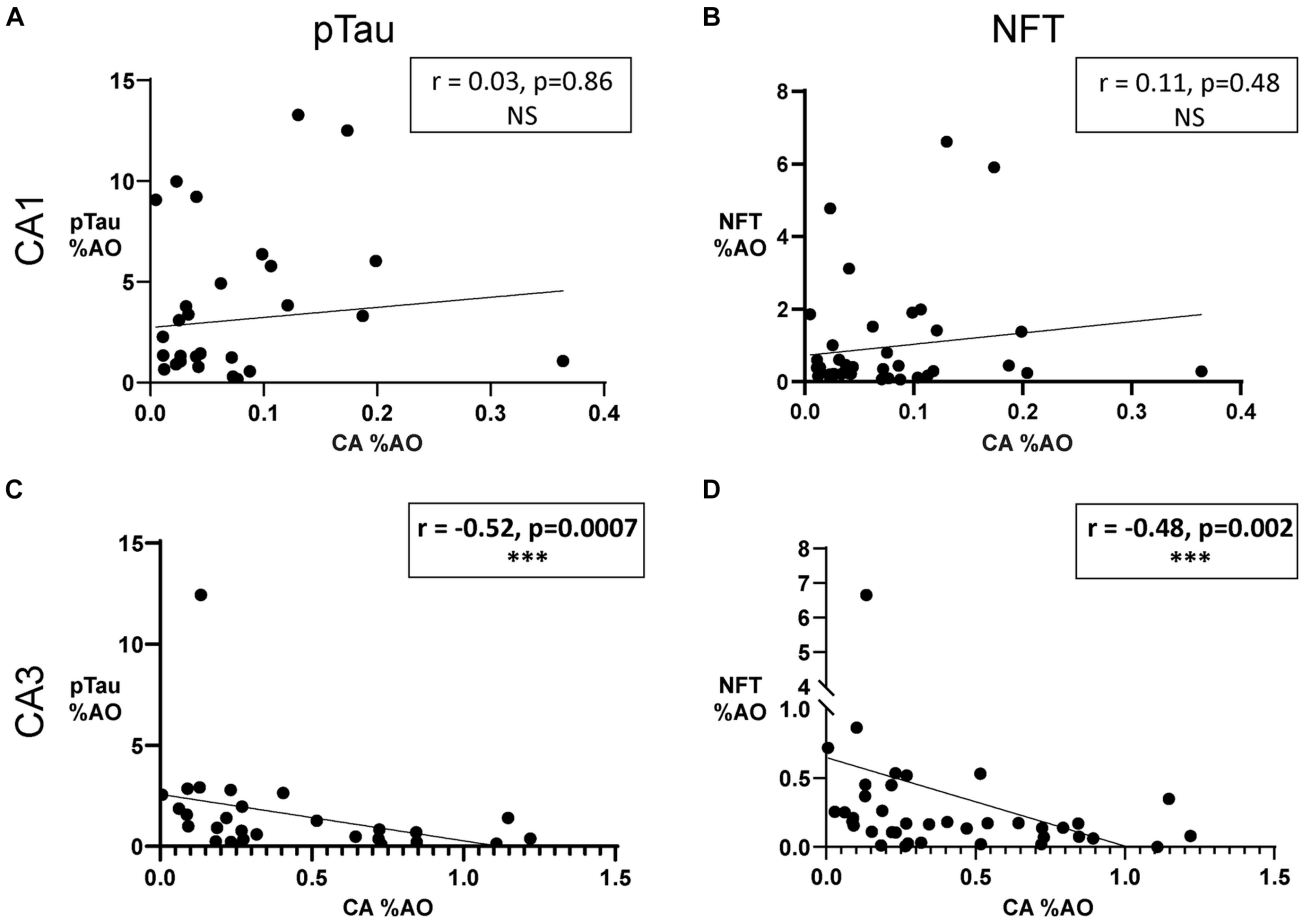
Amount of Corpora Amylacea is negatively associated with amount of NFTs in the CA3 region of donors with AD. **(A)** Scatter plot and line of best fit for pTau %AO vs. CA %AO in the CA1 hippocampal subregion. Spearman correlation revealed no significant relationship. **(B)** Scatter plot and line of best fit for NFT %AO vs. CA %AO in the CA1 hippocampal subregion. No significant association was found. **(C)** Scatter plot and line of best fit for pTau %AO vs. CA %AO in the CA3 hippocampal subregion. Spearman correlation revealed a significant negative correlation between the variables (*r* = –0.52, *p* = 0.0007). **(D)** Scatter plot and line of best fit for NFT %AO vs. CA %AO in the CA3 hippocampal subregion. A significant negative correlation was noted (*r* = –0.48, *p* = 0.002). Axis ranges varied by region and measurement and a discontinuous axis was used to visualize all data points.

Analysis of CA and tau in each subregion showed that CA %AO decreases with increasing pTau %AO (*r* = –0.52, *p* = 0.0007) and increasing NFT %AO (*r* = –0.48, *p* = 0.002) in the CA3 region (Figures 5C,D). CA was not significantly correlated with either pTau (Figure 5A) or NFT (Figure 5B) in the CA1 region of AD. Sensitivity analysis revealed no significant change when removing statistical outliers.

### 3.5 Amount of corpora amylacea is not associated with genetic risk score in AD or unaffected brain donors

Our previous study (Wander et al., 2022) found an inconsistent relationship between CA and *APOE* genotype across Braak stages. This study looked at the association of *APOE* e4 carrier status (*APOE* e4 carriers vs. non-carriers) with CA in the CA1 and CA3 regions of AD and unaffected control brains. We performed a non-parametric Mann–Whitney test to determine if CA %AO varied based on *APOE* e4 carrier status. No significant difference was found between *APOE* e4 carriers and non-carriers in the CA1 region of AD brain donors (*p* = 0.1) or unaffected controls (*p* = 0.7). Additionally, no significant difference was found in the CA3 region of AD brain donors (*p* = 0.6) or unaffected controls (*p* = 0.9).

**Figure 6:**
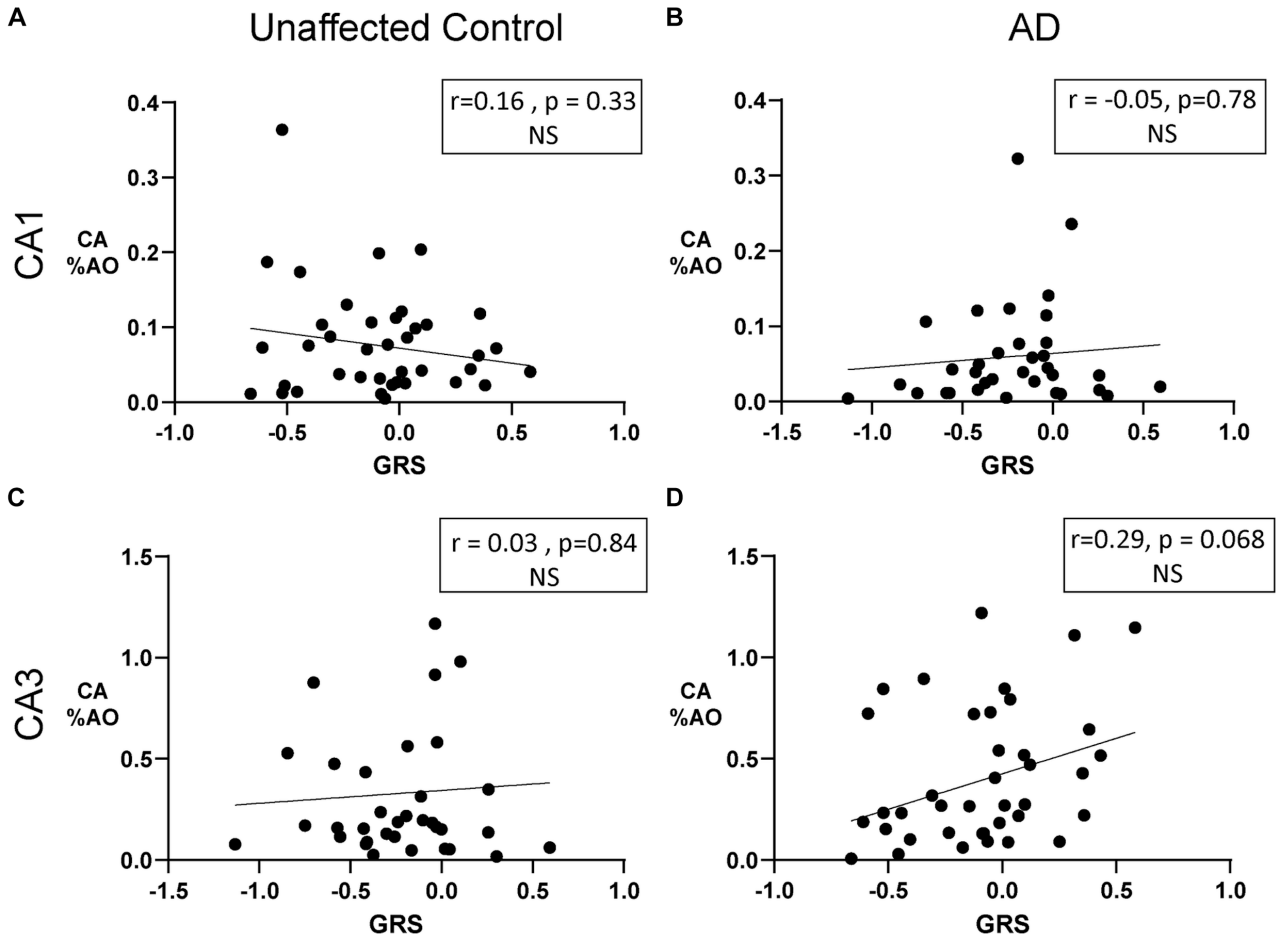
Amount of Corpora Amylacea is not associated with genetic risk in donors with AD or unaffected controls. **(A)** Scatter plot of CA %AO with GRS in the CA1 region of unaffected donors. **(B)** Scatter plot of CA %AO with GRS in the CA1 region of AD brain donors. No significant relationship was noted. **(C)** Scatter plot of CA %AO with GRS in the CA3 region of unaffected brain donors. **(D)** Scatter plot of CA %AO with GRS in the CA3 region of AD brain donors. Black line represents the line of best fit for the Pearson correlations. Note: Axis ranges varied by region and disease status to visualize all data points.

To further explore the effect of AD genetic risk, we investigated the relationship between a genetic risk score (GRS) and CA %AO. As seen in Figure 6, no correlations were noted in the CA1 (Figure 6A; *r* = 0.16, *p* = 0.33) and CA3 (Figure 6C; *r* = 0.03, *p* = 0.84) from unaffected controls (Figures 6A,C). Similarly, no correlations between GRS and CA %AO were found in the CA1 (Figure 6B; *r* = –0.05, *p* = 0.78) or CA3 (Figure 6D; *r* = 0.29, *p* = 0.068) region of AD brain donors. Overall, there does not appear to be an association of GRS or *APOE* on CA %AO.

### 3.6 Amount of corpora amylacea in the CA3 region of the hippocampus of AD donors is independently associated with the amount of pTau and age

The univariate results indicated the need to simultaneously investigate the effects of age and hippocampal region, which are associated with CA %AO. We employed a stepwise generalized linear model (GLM) to investigate these relationships in a mutually adjusted model. The CA3 region was the major focus due to previous univariate results. A backward selection process was used, and variables were excluded based on changes in AIC value (Supplementary Table S1).

The model focused solely on AD brain donors and consisted of the area occupied by CA as the dependent variable. It included %AO by pTau, age at death, sex, GRS, and *APOE* e4 allele count as the independent variables. The stepwise model excluded sex, GRS, and *APOE* due to significant increases in AIC, which can be seen in Supplementary Table S1. The GLM in the CA3 region detected a significant association between CA %AO and pTau %AO (beta = –0.05, *p* = 0.02) (Table 1). Age was also significantly associated with CA (beta = 0.01, *p* = 0.02).

**Table 1:** Stepwise generalized linear regression model results representing CA %AO by pTau %AO and age.

Predictors	Beta	*P*-value
pTau %AO	–0.05	0.02*
Age	0.16	0.02*

Sex, GRS, and APOE were excluded in the stepwise process. The model was fitted with a gamma distribution and a log link function. Statistical significance is indicated with a * (0.05 level).

## 4 Discussion

The results of this study support our hypothesis that CA act to reduce AD pathology by influencing pTau and NFT formation in the hippocampus. Neuropathological analysis of the hippocampus from AD brain donors and unaffected controls revealed associations between CA, pTau, and age in each group. While both groups exhibited higher levels of CA in the CA3 region, the relationships appeared to vary in the presence of pTau pathology. In the unaffected control group, we observed an elevation of CA %AO with age which is consistent with the literature (Martin et al., 1991; Cavanagh, 1999). However, in AD this relationship was absent in our univariate analysis. Multivariate analysis revealed that after accounting for the known effect of age, CA are negatively associated with pTau. This may suggest an accumulation of CA as we age, that is offset and reversed by the buildup of pTau that occurs in AD. These data support our hypothesis of pTau-influenced CA depletion and support a complex “tug-of-war” between age and AD pathological protein deposition that influence CA levels over time.

### 4.1 CA variation across hippocampal subregions

Significant differences were found between the CA1 and CA3 regions of the hippocampus in both AD and unaffected controls. Few studies have performed a quantitative analysis of CA across hippocampal regions. One study by Xu et al. (2021) investigated CA distribution in sleep apnea patients and found CA were most abundant in periventricular regions (alveus, wall of lateral ventricle, fimbria) and undetectable in the CA2 region. Xu et al. (2021) found few CA in the CA1 and CA3 regions. In our study, CA3 exhibited a higher area occupied by CA than CA1. Understanding the accumulation patterns of CA in the hippocampus will help identify CA extrusion sites and further reveal information regarding the kinetics of *CA.* As seen in this study, our results may suggest that the CA1 region is not a major CA aggregation site in brain donors with AD or in unaffected controls. The CA3 region appears to have a significantly higher area occupied by CA in both AD and unaffected control brains. This supports the hypothesis that CA are extruded into the CSF near the CA3 region under normal conditions. The process of CA extrusion has been outlined in detail by Riba et al. (2019) CA distribution patterns may also vary in different disease states due to disease-specific waste deposition. The presence and relevance of CA in disease states have been investigated in amyotrophic lateral sclerosis (ALS), Parkinson’s disease (PD), and vascular dementia (VaD), suggesting broad disease relevance (Pisa et al., 2016). Variability in hippocampal sectioning can alter the size and number of pathological findings. We controlled these sources of variation by using anatomical landmarks and a %AO formula to consider the ROI area and the size of stained features. Examination of additional brain regions affected by AD may reveal additional patterns of CA accumulation and may help illuminate the underlying mechanisms.

### 4.2 CA accumulation with age

Previous research has demonstrated that corpora amylacea increase with age (Cavanagh, 1999) in the spinal cord and anterior horn gray matter. This is likely due to accumulated waste, metabolic byproducts, and pathological debris over time. This accumulation with age could also signify a dysfunction of CA clearance, leading to aggregations of CA in various brain regions. In this study, we found a significant, positive correlation of CA with age in the CA3 region of the hippocampus of unaffected donors. The correlation in AD brain donors was similar but did not reach statistical significance. Interestingly, we did not find any significant correlations in the CA1 region in either group. Research in the spinal cord has found a linear increase in CA with age (Sakai et al., 1969). This association may be region and tissue specific. The relationship between CA accumulation and age may vary across the nervous system and may be limited due to the range of ages used in this study. Although no significant correlation was found between age and CA area occupied in donors with AD, the correlation coefficient and *p* value were similar to that of the correlation with unaffected donors. The minor reduction in significance and coefficient may be explained by sample size, cohort variation, or may suggest a slight disruption of the amount of CA in the presence of pTau aggregation. Additionally, in our multivariable analysis, after controlling for amount of pTau, age appeared to be a significant predictor of CA in the CA3 region of AD donors. Additional studies are needed assess the robustness of the association of Age and CA accumulation in the context of AD.

### 4.3 Genetic influence on CA amount

Several factors can influence NFT formation, including genetic variation. One study aimed to predict Braak stage antemortem and found that *APOE* e4 allele presence, age, high total cholesterol, and poor scores on neuropsychological tests could predict post-mortem Braak stage (Carlson et al., 2018). Various genetic factors have been shown to influence AD pathology. Gomez-Isla et al. found that some *PSEN1* mutations were associated with faster rates of NFT formation (Gómez-Isla et al., 1999). The presence of *APOE* e4 has also been associated with the degree of NFT formation (Kumar, 2022). Additionally, *APOE* e2/e3 genotype carriers exhibited lower NFT densities than *APOE* e3/e3 homozygotes (Lippa et al., 1997). If the genetic risk for AD influences AD pathology, it may also influence *CA.*

Our previous research (Wander et al., 2022) found that symptomatic tau transgenic and *APOE* knock-in mice exhibited higher densities of PAS granules, the mouse equivalent of *CA.* Additionally, among Braak NFT stage II donors, carriers of the *APOE* e3/e4 genotype had lower levels of CA in the dentate gyrus compared to *APOE* e3/e3 genotype carriers. In this study, we further investigated the role of *APOE* by investigating other hippocampal regions (CA1 and CA3) and by including *APOE* e4 allele count in our univariate and regression analysis. Interestingly, we found no association between CA %AO and *APOE* e4 carrier status in the CA1 or CA3 hippocampal regions of AD brain donors or unaffected controls. The difference between our two studies is likely due to the different distribution of Braak NFT stages. The current study consisted mainly of AD donors with late-stage disease (Braak NFT stage IV-VI) and unaffected controls with low pathology levels (Braak NFT stage 0-II). Our univariate analysis found no significant correlation between CA %AO and GRS in the CA3 or CA1 regions of AD brain donors. Although insignificant, a strong positive trend was found between CA amount and GRS in the CA3 region of the hippocampus in AD brain donors. Genetic risk variants such as those in *TREM2* (Replogle et al., 2015), *PICALM* (Ando et al., 2022), and *BIN1* (Chapuis et al., 2013), which are included in the GRS, are associated with various aspects of waste clearance. This raises questions about the possibility that genetic factors may influence CA activity. However, such variants are rare, and the effect of these variants is likely outweighed by the number of variants included in the GRS model. Additionally, the correlation weakened when adjusted for age and tau pathology, suggesting that the relationship is potentially due to the interaction between GRS and tau. These data suggest a Braak stage-dependent interaction between genetic risk and CA size and number, which requires additional investigation.

### 4.4 Association between amount of CA and pTau or NFT

In AD, pTau and amyloid beta accumulate progressively across various brain regions, with the hippocampus as a major aggregation site. Within the hippocampus, pTau accumulates in the entorhinal cortex and CA1 in early disease, where it is thought to eventually spread to the CA2 and CA3 regions later in the disease process (Braak and Braak, 1995; De Calignon et al., 2012) The location and density of NFTs may be associated with the cognitive dysfunction seen in AD. Consistent with previous research (Mizutani and Shimada, 1991; Kril et al., 2002), we found more pTau and NFTs in the CA1 region compared to the CA3 region. Corpora amylacea may modify levels of pTau, which in turn could influence NFT pathology over time. Corpora amylacea are generated by astrocytes in response to excess cellular and metabolic waste products (Riba et al., 2019). Previous studies have identified tau inside CA (Wander et al., 2022; Riba et al., 2023), specifically tau hypo-phosphorylated at the tau-1 epitope (S202 and T205) and total tau (tau-5). To date, no studies have specifically demonstrated hyperphosphorylated tau in CA; this highlights the need to examine how CA react to different tau isoforms, specifically pathogenic isoforms found in AD, such as hyperphosphorylated tau. CA containing tau highlights a potential clearance mechanism where CA may buffer pTau aggregation by sequestering smaller tau aggregates and extruding themselves into the CSF.

Colocalization was investigated by using the PAS-AT8 double stains. After visual inspection of the 78 brain samples in this study, there appeared to be common aggregation patterns. CA aggregated mainly near the ventricular lining and near damaged blood vessels. This is consistent with the hypothesized extrusion process in which CA are exported into the ventricles. In contrast, NFTs and pTau generally formed in the neuronal layers, specifically the pyramidal neuronal later of the hippocampus. CA were occasionally seen in the neuronal layers. Overall double staining revealed little evidence of colocalization at advanced stages of AD pathology.

Our previous research found a quadratic distribution of CA across Braak NFT stage in the dentate gyrus, with a peak at stage III, followed by a steady decline (Wander et al., 2022). In this study, we aimed to expand our findings by exploring other biologically relevant hippocampal regions and by using a quantitative metric of both CA and NFTs. We identified a negative relationship between CA and pTau %AO in the CA3 region of the hippocampus of AD brain donors, but no relationship in the CA1 region. The negative association between CA and both pTau %AO and NFT %AO may suggest the accumulation of pTau and NFT leads to a reduction in CA number, or a reduction in CA number leads to accumulation of pTau and NFT formation in AD. Alternatively, a third unknown variable may be impacting both, as there is no direct evidence of causality in these cross-sectional data. It is still unknown whether CA arise in response to pTau or pTau formation hinders CA production. It is also possible that an unidentified factor may be contributing to both processes.

CA appear to function in conjunction with various homeostatic pathways in the brain that prevent buildup of toxic waste, including AD-associated pathology. The Autophagy lysosome pathway (ALP), Ubiquitin proteosome system (UPS), CA, and the glymphatic system have all been implicated in AD, specifically with dysfunction of these systems leading to higher levels of AD pathology. These mechanisms also appear to interact with one another. For example, CA have been shown to contain ubiquitin, providing evidence that they may interact with the UPS, but more research is needed to elucidate their relationship (Augé et al., 2018). Additionally, the glymphatic system may be associated with the extrusion of CA, since CA are thought to be a hallmark of chronic glymphatic insufficiency (Riba et al., 2022b). It is likely these waste clearance mechanisms work together to identify, sequester, and clean the brain from pathological debris, and in proteinopathies such as AD, their proper functioning is essential to prevent or delay disease progression.

The use of cross-sectional studies on post-mortem brain tissue limits cause-effect conclusions and renders it difficult to understand the temporal aspect of this association. To partially address this, we used brain regions affected in early and late-stage AD; however, more research is needed to understand the temporal aspect of the relationship between pTau and *CA.* Our results vary across hippocampal regions, adding to the complexity of the interpretation of results. Regardless, our data are in line with the results from our previous research from Wander et al. (2022), and support the idea that CA play a role in AD pathogenesis.

## 5 Conclusion

Overall, results from this study provide evidence of a relationship between pTau and CA, with CA potentially playing a role in AD pathogenesis by clearing pTau and delaying the formation of NFTs; however, more research is needed to determine the sequence of events leading to this association. If CA can sequester and expel tau from the brain, increased CA activity could increase resistance to AD neuropathology and prove to be a potential therapeutic target for the prevention or treatment of AD pathology buildup.

## Data availability statement

The raw data supporting the conclusions of this article will be made available by the authors, without undue reservation.

## Ethics statement

The studies involving humans were approved by University of Miami Institutional Review Board (IRB) protocol 19920348. The studies were conducted in accordance with the local legislation and institutional requirements. The human samples used in this study were acquired from Existing samples from the University of Miami Brain Endowment Bank. Written informed consent for participation was not required from the participants or the participants’ legal guardians/next of kin in accordance with the national legislation and institutional requirements.

## Author contributions

JD: Conceptualization, Data curation, Funding acquisition, Investigation, Methodology, Writing – original draft, Writing – review & editing, Formal analysis, Project administration. RG: Data curation, Formal analysis, Investigation, Methodology, Writing – review & editing. RV: Investigation, Methodology, Supervision, Writing – review & editing. AB: Data curation, Investigation, Methodology, Writing – review & editing. DDo: Data curation, Formal analysis, Methodology, Writing – review & editing. DDa: Investigation, Methodology, Supervision, Writing – review & editing. XS: Methodology, Supervision, Writing – review & editing. DB: Data curation, Methodology, Writing – review & editing. IB: Data curation, Writing – review & editing. SG: Methodology, Writing – review & editing. CW: Conceptualization, Methodology, Writing–review & editing. TC: Conceptualization, Supervision, Writing – review & editing. WS: Conceptualization, Investigation, Resources, Supervision, Writing – review & editing.
