# Supplementary material for: Corpora amylacea negatively correlate with hippocampal tau pathology in Alzheimer’s disease
Source: Front Neurosci. 2024 Feb 29;18:1286924. doi: 10.3389/fnins.2024.1286924 (PMC10937356; doi:10.3389/fnins.2024.1286924)
Supplement: Supplementary file 1 [file Table_1.DOCX]

**Supplementary Data**

**Supplementary Table 1.** Stepwise Regression Model Building for CA ~ pTau + GRS + *APOE* + Age + Sex

| Model Step | Model Variables | Removed Variable | AIC |
| --- | --- | --- | --- |
| Start | pTau CA3, GRS, *APOE*, Age, Sex | None | -83.97 |
| Step 1 | pTau CA3, GRS, *APOE*, Age | Sex | -85.94 |
| Step 2 | pTau CA3, *APOE*, Age | GRS | -86.97 |
| Final Model | pTau CA3, Age | *APOE* | -88.5 |
