# Supplementary material for: Corpora amylacea negatively correlate with hippocampal tau pathology in Alzheimer’s disease
Source: Front Neurosci. 2024 Feb 29;18:1286924. doi: 10.3389/fnins.2024.1286924 (PMC10937356; doi:10.3389/fnins.2024.1286924)
Supplement: Supplementary file 2 [file Table_2.DOCX]

**Supplementary Data**

**Supplementary Table 2.** Detailed Demographics of Unaffected Control Brain Donors

| **Code** | **Clinical Assessment** | **PMI** | **Brain Weight (g)** | **Age** | **Race** | **Sex** | **COD** | **B** | **Braak** |
| --- | --- | --- | --- | --- | --- | --- | --- | --- | --- |
| **1** | Unaffected Control | 3.75 | 1162 | 85 | White non-Hispanic | female | Metastatis lung cancer | 0 | Stage 0 |
| **2** | Unaffected Control | 15 | 1200 | 90+ | White non-Hispanic | male | Biventricular systolic heart failure | 1 | Stage I |
| **3** | Unaffected Control | 12 | 1289 | 90+ | White non-Hispanic | female | COPD | 1 | Stage I |
| **4** | Unaffected Control | 11.5 | 1250 | 90+ | White non-Hispanic | male | Coronary artery disease, HTN, Bladder cancer | 2 | Stage III |
| **5** | Unaffected Control | 8.3 | 1002 | 90+ | White non-Hispanic | female | Multiple diseases of the aged | 1 | Stages I-II |
| **6** | Unaffected Control | 15.58 | 1275 | 85 | White non-Hispanic | male | Multiple sclerosis | 1 | Stages I-II |
| **7** | Unaffected Control | 20.5 | 1295 | 90+ | White non-Hispanic | male | End stage CHF | 0 | Stage 0 |
| **8** | Unaffected Control | 5.1 | 1027 | 90+ | White non-Hispanic | female | Cerebral infarct, Arteriosclerotic cardiovascular disease | 0 | Stage 0 |
| **9** | Unaffected Control | 10 | 1136 | 90+ | White non-Hispanic | female | Lung carcinoma, metastatic | 0 | Stage 0 |
| **10** | Unaffected Control | 5 | 1057 | 90+ | White non-Hispanic | female | CHF, Dilated cardiomyopathy, ASHD | 0 | Stage 0 |
| **11** | Unaffected Control | 23 | 1131 | 90+ | White non-Hispanic | female | Metastatis adenocarcinoma | 0 | Stage 0 |
| **12** | Unaffected Control | 4 | 1071 | 85 | White non-Hispanic | female | Cardiopulmonary arrest, Respiratory failure | 0 | Stage 0 |
| **13** | Unaffected Control | 4.75 | 1321 | 90+ | White non-Hispanic | male | Acute respiratory failure, CHF | 0 | Stage 0 |
| **14** | Unaffected Control | 2.5 | 1050 | 90+ | White non-Hispanic | female | Acute MI, CAD, HTN, CHF | 0 | Stage 0 |
| **15** | Unaffected Control | 7.5 | 1569 | 82 | White non-Hispanic | male | Gallbladder cancer with metastisis to stomach | 0 | Stage 0 |
| **16** | Unaffected Control | 12 | 1196 | 90+ | White non-Hispanic | female | CHF | 0 | Stage 0 |
| **17** | Unaffected Control | 3 | 1150 | 78 | White non-Hispanic | female | Multiple myeloma, CHF | 0 | Stage 0 |
| **18** | Unaffected Control | 8 | 1194 | 87 | White non-Hispanic | female | Acute myeloid leukemia | 0 | Stage 0 |
| **19** | Unaffected Control | 9 | 1150 | 89 | White non-Hispanic | female | Renal insufficiency, CAD, CHF | 0 | Stage 0 |
| **20** | Unaffected Control | 28 | 1350 | 65 | White non-Hispanic | male | Electrocution | 0 | Stage 0 |
| **21** | Unaffected Control | 5.5 | 967 | 87 | White non-Hispanic | female | Lung cancer w/metastasis | 0 | Stage 0 |
| **22** | Unaffected Control | 3.15 | 1063 | 90+ | White non-Hispanic | female | CHF, ASHD | 0 | Stage 0 |
| **23** | Unaffected Control | 17 | 1147 | 87 | White non-Hispanic | female | Respiratory failure, Hypertension | 0 | Stage 0 |
| **24** | Unaffected Control | 21 | 1375 | 87 | White non-Hispanic | male | Cardiac arrest, CAD | 0 | Stage 0 |
| **25** | Unaffected Control | 7.3 | 1200 | 89 | White non-Hispanic | male | Acute MI, CHF Acute, GI Bleeding | 0 | Stage 0 |
| **26** | Unaffected Control | 12.25 | 1256 | 84 | White non-Hispanic | female | Cardiac arrhymia, coronary artery disease | 0 | Stage 0 |
| **27** | Unaffected Control | 7.33 | 1294 | 85 | White non-Hispanic | male | Ischemic cardiomyopathy, CAD | 0 | Stage 0 |
| **28** | Unaffected Control | 8.5 | 1220 | 70 | White non-Hispanic | male | Cardiac arrest, Acute MI | 0 | Stage 0 |
| **29** | Unaffected Control | 22 | 1430 | 67 | White non-Hispanic | male | Atherosclerotic heart disease | 0 | Stage 0 |
| **30** | Unaffected Control | 18 | 1200 | 74 | White non-Hispanic | male | Pancreatic cancer | 0 | Stage 0 |
| **31** | Unaffected Control | 17.5 | 1436 | 90+ | White non-Hispanic | male | Cardiopulmonary arrest, coronary artery disease | 0 | Stage 0 |
| **32** | Unaffected Control | 19 | 1460 | 67 | White non-Hispanic | male | Myocardial infarction, Coronary artery atherosclerosis | 0 | Stage 0 |
| **33** | Unaffected Control | 8.5 | 1710 | 67 | White non-Hispanic | male | MI | 0 | Stage 0 |
| **34** | Unaffected Control | 26 | 1330 | 65 | White non-Hispanic | male | Occulsive coronary artery, Coronary atherosclerosis | 0 | Stage 0 |
| **35** | Unaffected Control | 19.5 | 1460 | 64 | White non-Hispanic | male | Atherosclerotic heart disease | 0 | Stage 0 |
| **36** | Unaffected Control | 5 | 984 | 87 | White non-Hispanic | female | Cardiopulmonary arrest, coronary artery disease | 0 | Stage 0 |
| **37** | Unaffected Control | 3.25 | 1260 | 88 | White non-Hispanic | female | End stage COPD | 0 | Stage 0 |
| **38** | Unaffected Control | 17.25 | 1500 | 65 | White non-Hispanic | male | Critical coronary artery atherosclerosis, Hypertension | 0 | Stage 0 |

*Note:* PMI = postmortem interval; B Score = Neurofibrillary tangles (B score collapses Braak stages: B1 = Braak I-II, B2 = Braak III-IV, B3 = Braak V-VI); Braak = Braak and Braak Staging of NFT location and density; COD = Cause of Death; AD = Alzheimer’s disease.
