# Supplementary material for: Corpora amylacea negatively correlate with hippocampal tau pathology in Alzheimer’s disease
Source: Front Neurosci. 2024 Feb 29;18:1286924. doi: 10.3389/fnins.2024.1286924 (PMC10937356; doi:10.3389/fnins.2024.1286924)
Supplement: Supplementary file 3 [file Table_3.DOCX]

**Supplementary Data**

**Supplementary Table 3.** Detailed Demographics of Human AD Brain Donors

| **AD** | **Clinical Assessment** | **PMI** | **Brain_wt** | **Age** | **Race** | **Sex** | **COD** | **B** | **Braak** |
| --- | --- | --- | --- | --- | --- | --- | --- | --- | --- |
| **1** | AD | 20.41 | 1100 | 90+ | White non-Hispanic | female | Cornoary Artery Disease | 3 | Stage V-VI |
| **2** | AD | 28.2 | 1100 | 88 | White non-Hispanic | male | Dementia | 3 | Stage V-VI |
| **3** | AD | 12.23 | 1000 | 90+ | White non-Hispanic | female | Cardiorespiratory arrest, Cerebral vascular accident | 3 | Stage V-VI |
| **4** | AD | 8.51 | 1075 | 83 | White non-Hispanic | female | COPD | 3 | Stage V-VI |
| **5** | AD | 20.5 | 1200 | 60 | White non-Hispanic | female | Alzheimer's Disease | 3 | Stage V-VI |
| **6** | AD | 6.1 | 1275 | 84 | White non-Hispanic | male | Sepsis, UTI, Advanced Dementia | 3 | Stage V-VI |
| **7** | AD | 3 | 1328 | 63 | White non-Hispanic | male | Cardiopulmonary Arrest | 3 | Stage V-VI |
| **8** | AD | 17.61 | 1250 | 74 | White non-Hispanic | female | Alzheimer's Disease | 3 | Stage VI |
| **9** | AD | 3.88 | 922 | 83 | White non-Hispanic | female | Senile degeneration of the brain with dementia | 3 | Stage VI |
| **10** | AD | 22 | 990 | 70 | White non-Hispanic | female | Chronic debility, senile degeneration of the brain | 3 | Stage VI |
| **11** | AD | 8 | 1070 | 70 | White non-Hispanic | male | Cerebral Atherosclerosis, dementia | 3 | Stage VI |
| **12** | AD | 5.73 | 1200 | 76 | White non-Hispanic | female | Natural cuase, Cerebrovascular Disease | 3 | Stage VI |
| **13** | AD | 6.65 | 1238 | 84 | White non-Hispanic | male | Alzheimer's Disease | 3 | Stage VI |
| **14** | AD | 4.5 | 1051 | 81 | White non-Hispanic | female | Cerebral Atherosclerosis | 3 | Stage VI |
| **15** | AD | 10.06 | 1029 | 75 | White non-Hispanic | male | Cardiopulmonary arrest | 3 | Stage VI |
| **16** | AD | 7.29 | 1025 | 75 | White non-Hispanic | male | Atherosclerosis | 3 | Stage VI |
| **17** | AD | 15 | 841 | 63 | White non-Hispanic | female | Alzheimer's Disease | 3 | Stage VI |
| **18** | AD | 20.33 | 1126 | 88 | White non-Hispanic | female | Alzheimer's Disease | 3 | Stage VI |
| **19** | AD | 8.3 | 985 | 90+ | White non-Hispanic | female | Atherosclerotic Heart Disease | 3 | Stage VI |
| **20** | AD | 11.61 | 1040 | 71 | White non-Hispanic | male | Rapidly progressing cognitive decompensation | 3 | Stage VI |
| **21** | AD | 12.6 | 1137 | 89 | White non-Hispanic | male | Cardiopulmonary arrest | 3 | Stage VI |
| **22** | AD | 3.81 | 700 | 60 | White non-Hispanic | female | Congestive heart failure | 3 | Stage VI |
| **23** | AD | 17.3 | 1125 | 59 | White non-Hispanic | male | Alzheimer's Disease | 3 | Stage VI |
| **24** | AD | 26 | 1092 | 68 | White non-Hispanic | female | Alzheimer's Disease | 3 | Stage VI |
| **25** | AD | 20 | 860 | 67 | White non-Hispanic | male | Alzheimer's Disease | 3 | Stage VI |
| **26** | AD | 17.61 | 1168 | 87 | White non-Hispanic | male | Cerebrovascular disease, Alzheimer's Disease | 3 | Stage VI |
| **27** | AD | 18.5 | 850 | 59 | White non-Hispanic | female | Cardiopulmonary arrest, Respirator failure | 3 | Stage VI |
| **28** | AD | 22.5 | 1359 | 89 | White non-Hispanic | female | Alzheimer's Disease | 3 | Stage VI |
| **29** | AD | 26.66 | 1110 | 90+ | White non-Hispanic | female | Alzheimer's Disease | 3 | Stage VI |
| **30** | AD | 26 | 1100 | 85 | White non-Hispanic | male | Cerebral Degeneration | 3 | Stage VI |
| **31** | AD | 19 | 1107 | 55 | White non-Hispanic | female | Alzheimer's Disease | 3 | Stage VI |
| **32** | AD | 8.5 | 1005 | 84 | White non-Hispanic | female | Alzheimer's Disease | 3 | Stage V |
| **33** | AD | 7.21 | 1132 | 90+ | White non-Hispanic | male | Cardiopulmonary Arrest | 2 | Stage IV |
| **34** | AD | 12 | 1025 | 90+ | White non-Hispanic | female | Alzheimer's Disease | 2 | Stage IV |
| **35** | AD | 18.28 | 1050 | 79 | White non-Hispanic | female | Acute and chronic  Respiratory failure | 2 | Stage III |
| **36** | AD | 15 | 930 | 90+ | White non-Hispanic | female | Cerebral Infarction due to Thrombosis | 2 | Stage III |
| **37** | AD | 19.4 | 875 | 61 | White non-Hispanic | female | Alzheimer's Disease | 2 | Stage IV |
| **38** | AD | 7.35 | 1064 | 90+ | White non-Hispanic | female | Dementia | 2 | Stage IV |
| **39** | AD | 5.5 | 963 | 78 | White non-Hispanic | female | Septicemia | 3 | Braak V-VI |
| **40** | AD | 7.35 | 1075 | 90+ | White non-Hispanic | female | Cardiopulmonary Arrest | 3 | Braak V-VI |

*Note:* PMI = postmortem interval; B Score = Neurofibrillary tangles (B score collapses Braak stages: B1 = Braak I-II, B2 = Braak III-IV, B3 = Braak V-VI); Braak = Braak and Braak Staging of NFT location and density; COD = Cause of Death; AD = Alzheimer’s disease.
